# Engagement of intrinsic disordered proteins in protein–protein interaction

**DOI:** 10.3389/fmolb.2023.1230922

**Published:** 2023-07-31

**Authors:** Irena Roterman, Katarzyna Stapor, Leszek Konieczny

**Affiliations:** ^1^ Department of Bioinformatics and Telemedicine, Jagiellonian University—Medical College, Kraków, Poland; ^2^ Department of Applied Informatics, Faculty of Automatic, Electronics and Computer Science, Silesian University of Technology, Gliwice, Poland; ^3^ Chair of Medical Biochemistry, Medical College, Jagiellonian University, Kraków, Poland

**Keywords:** intrinsically disordered proteins, disport, hydrophobicity, protein complex, function-related structural changes, order-to-disorder, MoRF IR

## Abstract

Proteins from the intrinsically disordered group (IDP) focus the attention of many researchers engaged in protein structure analysis. The main criteria used in their identification are lack of secondary structure and significant structural variability. This variability takes forms that cannot be identified in the X-ray technique. In the present study, different criteria were used to assess the status of IDP proteins and their fragments recognized as intrinsically disordered regions (IDRs). The status of the hydrophobic core in proteins identified as IDPs and in their complexes was assessed. The status of IDRs as components of the ordering structure resulting from the construction of the hydrophobic core was also assessed. The hydrophobic core is understood as a structure encompassing the entire molecule in the form of a centrally located high concentration of hydrophobicity and a shell with a gradually decreasing level of hydrophobicity until it reaches a level close to zero on the protein surface. It is a model assuming that the protein folding process follows a micellization pattern aiming at exposing polar residues on the surface, with the simultaneous isolation of hydrophobic amino acids from the polar aquatic environment. The use of the model of hydrophobicity distribution in proteins in the form of the 3D Gaussian distribution described on the protein particle introduces the possibility of assessing the degree of similarity to the assumed micelle-like distribution and also enables the identification of deviations and mismatch between the actual distribution and the idealized distribution. The FOD (fuzzy oil drop) model and its modified FOD-M version allow for the quantitative assessment of these differences and the assessment of the relationship of these areas to the protein function. In the present work, the sections of IDRs in protein complexes classified as IDPs are analyzed. The classification “disordered” in the structural sense (lack of secondary structure or high flexibility) does not always entail a mismatch with the structure of the hydrophobic core. Particularly, the interface area, often consisting of IDRs, in many analyzed complexes shows the compliance of the hydrophobicity distribution with the idealized distribution, which proves that matching to the structure of the hydrophobic core does not require secondary structure ordering.

## 1 Introduction

In the organization of a living organism, proteins perform a variety of functions. They can even be described as tools for the implementation of specific tasks. It is difficult to refer to a specific publication in this area. This topic is discussed collectively in numerous reviews (Alberts et al., 2002; Pazos and Sternberg, 2004; Laskowski et al., 2005; Bandyopadhyay et al., 2006; Shin et al., 2007; Redfern et al., 2009; Mills et al., 2015; Das and Orengo, 2016; Berezovsky et al., 2017a; Berezovsky et al., 2017b; Kuhlman and Bradley, 2019; Mak and Thurston, 2021). The sources of information on the structural and functional diversity are reports from centers dealing with the prediction of protein structure on the basis of the amino acid sequence (Roy et al., 2010; Khoury et al., 2014; Yang et al., 2015; Yang and Zhang, 2015; Kandathil et al., 2018; Yang et al., 2020). In the field of protein structure prediction, methods based on deep learning techniques play an important role (Zhang and Shen, 2020; Jumper et al., 2021; Service, 2021). This research is accompanied by the development of experimental techniques allowing for the identification and tracking of the process of protein synthesis and folding with the accuracy of a single amino acid (Henry Brinkerhoff et al., 2021; Ruff and Pappu, 2021; Serpell et al., 2021). Due to the high complexity of the system operating in the cell (and thus in a more organized organism), the need for various tools is also very high (Hellner et al., 2020). Systems Biology tries to provide solutions to the puzzle of the organization of a living organism, where the search for mutual relations is the basis for the construction of the organization of a complex system (Marcus, 2008; Konieczny et al., 2016; Lecca et al., 2016).

Constructing a comprehensive system illustrating the functioning of a living organism, taking into account evolutionary changes, becomes possible due to a large amount of data, including genetic and structural data (Johannes et al., 2020). The fundamental question of the mechanism by which the protein folding process takes place, however, remains open. In research on the structure of proteins, proteins known as intrinsically disordered (IDPs) and those in which a specific fragment exhibits structural variability (intrinsically disordered regions, IDRs) occupy a special place. The criterion used for their identification is the absence of secondary structure and the high flexibility that makes it impossible for X-ray techniques to determine the structure of a given segment.

The analysis of these proteins is presented in the form of extensive literature records (Oldfield et al., 2007; Dunker et al., 2008; Uversky and Dunker, 2010; Katuwawala et al., 2019), tools for IDR identification (Dyson and Wright, 2002; Obradovic et al., 2003; Vucetic et al., 2003; Xue et al., 2010; DISPROT, 2022; PONDR, 2023a), and numerous databases (Peng et al., 2005; Oldfield et al., 2008; Oates et al., 2013; Miskei et al., 2017; Necci et al., 2021; Quaglia et al., 2022a; Quaglia et al., 2022b; PONDR, 2023b; DISPROT, 2023).

The proteins analyzed in the present study were taken from the publication by Dunker et al. (2008) and partially from the work by Uversky and Dunker (2010). Here, an alternative evaluation of these proteins is proposed using the presence of a hydrophobic core and the degree of participation of IDRs in the construction of a common hydrophobic core in the complexes formed by representatives of IDPs. The evaluation used the fuzzy oil drop model (FOD) and its modified form (FOD-M), which was used to assess the specificity of other proteins, including membrane proteins (Dygut et al., 2016; Roterman et al., 2021a; Roterman et al., 2021b; Roterman et al., 2021c; Roterman et al., 2022a).

The hydrophobic core is treated as a factor stabilizing the tertiary structure. Therefore, the analysis of its participation in its structure in IDP proteins seems to be justified, especially if various biological functions require the use of unfolded or partially unfolded proteins. In the present work, examples of proteins analyzed in detail in Johannes et al. (2020) were used, proposing an alternative characterization of the structure of these proteins (Dunker et al., 2008). The results presented here complement the analyses carried out with the use of other methods, such as the PONDR method (Dyson and Wright, 2002; PONDR, 2023a) and the MoRF definition (Katuwawala et al., 2019). Complement is understood as the evaluation of the same proteins from a point of view different from that normally used in the evaluation of IDPs.

The applied model is based on the comparison between idealized hydrophobicity distribution expressed by the 3D Gauss function, T distribution (micelle-like distribution), and observed O based on the hydrophobic interaction between residues dependent on their intrinsic hydrophobicity and the distance between them. In quantitative measurements expressed by divergence entropy between T and O distributions, the divergence entropy is introduced (O|T). To make the entropy interpretable, the reference distribution R is introduced with an equal level of hydrophobicity, thus being opposite to the T distribution with a centric hydrophobic core. The RD (relative distance) expresses the O|T divergence entropy in relation to the sum of O|T and O|R. The RD < 0.5 identifies the proteins with the hydrophobic core present.

Since the water environment directing the folding process toward hydrophobic core generation is not the only one, to represent the influence of the hydrophobic surrounding in membrane proteins, the 1–3D Gauss function is introduced to represent the hydrophobicity distribution in membrane proteins. The influence of the other-than-water surrounding appears to be different. The degree of such influence is measured by the K parameter, which represents the level of lowering the polar water influence (Dygut et al., 2016; Roterman et al., 2021a; Roterman et al., 2021b; Roterman et al., 2021c; Roterman et al., 2022a).

## 2 Results

The interpretation of the RD and K parameter is as follows.

1. RD value characterizes the order of hydrophobicity distribution with respect to the idealized distribution expressed by the 3D Gauss function to represent the idealized micelle-like distribution of hydrophobicity—centric concentration of hydrophobicity (hydrophobic core) and polar surface. A *RD* = 0.5 is taken as the threshold. A RD value > 0.5 describes the status of the absence of a hydrophobic core.
2. K value assesses the degree to which the participation of other-than-water compounds directed the folding process. It is assumed that the environment directs this process toward an organization accordant to the external force field. *K = 0* characterizes the protein as representing a micelle-like organization directed by polar water. Proteins as such have been recognized: down-hill proteins, fast-folding proteins, ultra-fast-folding, and type II antifreeze proteins (Roterman et al., 2022a). The higher the K value, the higher the participation of external factors other than water. The membrane proteins acting in a hydrophobic environment are usually described by K > 1.0.

An alternative interpretation of the results of structural studies of complexes composed of proteins classified as IDPs or IDRs is presented here. This analysis may complement research on IDPs (Johannes et al., 2020). The proteins and their complexes presented in the present study are characterized by the RD and K parameters introduced by the FOD and FOD-M models. Descriptions of these models are included in Supplemental Materials. The value of RD < 0.5 means the arrangement of the hydrophobicity according to the arrangement of the centric hydrophobic core and the layer with reduced hydrophobicity is determined according to the 3D Gaussian distribution. The non-zero value of the K parameter in the FOD-M model denotes the degree of participation of factors other than the water polar environment, resulting in a local or comprehensive disturbance of the ideal distribution predicted by the FOD model. These parameters will be given for the complexes and individual chains included in the complex. The description of individual chains consists of two sets. One of them expresses the status of chains treated as individual structural units. This means that the 3D Gaussian function is spread over a single chain, identifying the presence of a hydrophobic core within it. In the second set, the given chain is treated as a component of the complex. In this case, the 3D Gauss function spans the whole complex, but the compatibility of the distribution of T and O applies only to a single chain. If a domain is present in the structure of a given chain, the status of the domain as an individual structural unit is also assessed.

The status of the interface area was also assessed. A RD value of <0.5 for this region indicates the alignment of the inter-chain interaction residues to the common core of the hydrophobic complex. It also means that hydrophobic interactions take part in the stabilization of a given complex.

The division into groups of analyzed proteins follows the division introduced in the publications of van der Lee et al. (2014) and Sharma et al. (2019).

### 2.1 Molecular recognition features (MoRFs)

The classification of complexes in which the sections recognized as IDR is involved according to the molecular recognition features (MoRFs) model which distinguishes the forms α-MoRF, β-MorF, and τ-MorF (van der Lee et al., 2014; Sharma et al., 2019). This classification emphasizes the preference to adopt a specific structure as a disordered protein (IDP) or its region (IDR) after fixation of the complex with the target molecule. The examples cited are as follows: saccharopepsin in complex with protease A inhibitor 3 (PDB—1DP5) (Li et al., 2000) representing the α-MORF group; protease from human adenovirus C serotype 2 in complex with pre-protein VI from human adenovirus C serotype 2 (PDB—1AVP) (Ding et al., 1996) representing β-MoRF, alpha-adaptin c—ap-2 clathrin adapter alpha subunit in complex with amphiphysin fxdx (PDB—1KY7) (Brett et al., 2002) representing τ-MORF characterized by the lack of a regular H-bonds system between target and complexed molecule, and phosphotyrosine-binding domain (PTB) of the X11 protein in complex with unphosphorylated peptides corresponding to a region of beta-amyloid precursor protein (beta-APP) (PDB—1X11) (Zhang et al., 1997). In this group of representatives, only 1X11 shows a structure with a hydrophobic distribution consistent with the 3D Gaussian distribution (Table 1). This correspondence exists in the structure of the complex as well as in the structural units of the complex, both as components of the complex and when treated as individual structural units. The remaining complexes and their components show a significant deviation from the structure based on stabilization resulting from the presence of a hydrophobic core. Only selected domains in 1DP5 and 1KY7 show micelle-like structuring. The interface status of 1K11 (β—amyloid precursor) and 1KY7 (τ-MoRF) shows local ordering consistent with the idealized hydrophobicity distribution. The interface status is also characterized by a low value, which proves the participation of this fragment of the complex in the structure of the hydrophobicity distribution common for the entire complex, which is characteristic of the present hydrophobic core. In the case of 1AVP (β-MoRF) and 1DP5 (α-MoRF), the interface status is expressed with high RD values, which means that the interface residues do not contribute to the construction of a common hydrophobic core (Table 1).

**TABLE 1:** Parameters assessing the status of the hydrophobic core in proteins that are examples of suitable groups within the MoRF classification.

PDB	Complex		Individual chains				Chains as a part of the complex				Interface	Characteristics
	RD	K	RD	K	RD	K	RD	K	RD	K	RD	
MoRF												
1X11	0.435	0.2	0.455	0.3	0.535	0.3	0.438	0.3	0.393	0.0	0.336	β-Amyloid precursor
1KY7	**0.649**	**0.8**	**0.648**	**0.7**	**0.712**	**1.5**	**0.654**	**0.8**	0.451	0.1	0.490	**D1: 0.625 (0.6)** and D2: 0.360 (0.2)
												τ-MoRF
1DP5	**0.616**	**0.8**	**0.618**	**0.8**	**0.704**	**1.1**	**0.611**	**0.8**	**0.651**	**0.7**	**0.693**	**D1: 0.549 (0.5)** and D2: 0.478 (0.3)
												D3: 0.454 (0.3) and D4: 0.391 (0.2)
												**E: 0.908**
												**SS: 0.628**; 0.287 (3Cat)
												α-MoRF
1AVP	**0.513**	**0.4**	**0.501**	**0.4**	**0.778**	**1.3**	**0.504**	**0.4**	**0.632**	**0.6**	**0.681**	β-MoRF

Dn—identification of domains; SS—fragment of chain limited by Cys positions constructing particular SS bonds.

**FIGURE 1:**
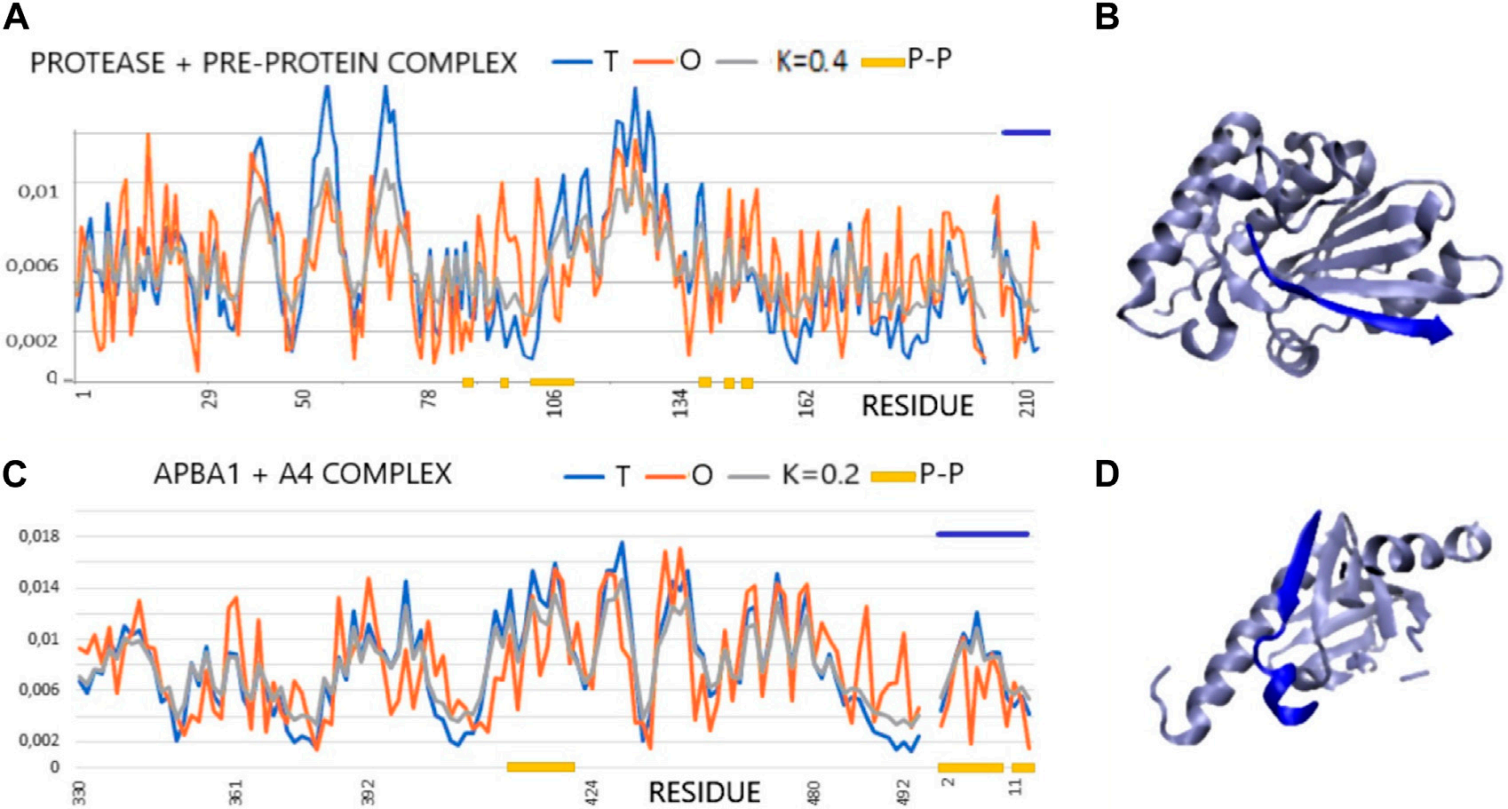
Profiles T, O, and M for the given K values for the complexes (x-axis: residues engaged in the P–P interaction—orange). **(A)** Protease and pre-protein complex (PDB ID—1AVP). **(B)** 3D presentation of protease and pre-protein complex. **(C)** ABPA1 in complex with β-amyloid precursor—A4. Chain A4 distinguished by the dark blue lines—top (PDB ID—1X11). **(D)** 3D presentation of ABPA1 in complex with β-amyloid precursor—A4 (dark blue).

The two examples of profiles given in Figures 1A, B illustrate examples of complexes with varying degrees of hydrophobic core order, although the RD determined for 1AVP slightly exceeds the cut-off level. On the other hand, 1X11 shows an order consistent with the idealized distribution in all assessments. There is also a highly consistent distribution of T and O within the peptide, which, in these two cases, adapted its structure to the system corresponding to the distribution of micelle-like hydrophobicity. The 3D presentation reveals the regular, globular structure of the proteins in question (Figure 1C).

### 2.2 Predictor of natural disordered regions (PONDR)

The second set of proteins are examples whose status under the IDP classification has been determined using an online tool called PONDR (Dyson and Wright, 2002; PONDR, 2023a). The analyzed proteins were taken from the work of Dunker et al. (2008). The use of this tool provides an assessment in the form of a predisposition profile of a given segment to the IDR status on the basis of the coefficient PONDR >0.5.

**FIGURE 2:**
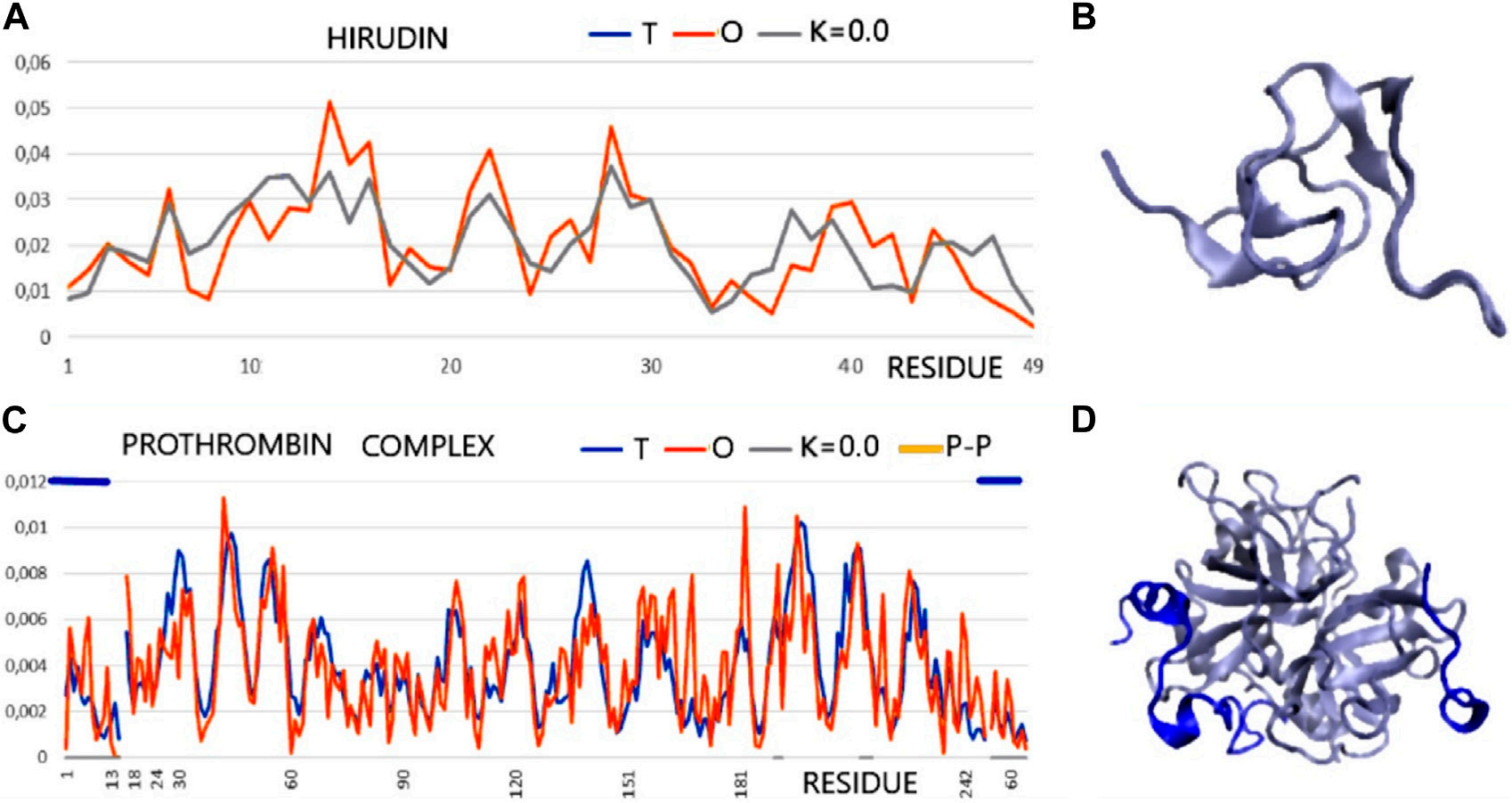
T and O profile (x-axis—positions of residues in the aa sequence in the chain, y-axis—hydrophobicity: T—blue, O—red). **(A)** Hirudin (PDB ID—5HIR). **(B)** 3D presentation of hirudin. **(C)** The prothrombin complex (PDB ID—1NO9) (*Homo sapiens*) (17–247—central part of profiles) with a fragment of prothrombin (1–16) and with a fragment of hirudin (C-terminal fragment—*Hirudo medicinalis*) distinguished by the dark blue lines—top axis. The positions involved in the construction of the interface are marked on the horizontal axis (bottom). **(D)** 3D presentation of 1N09—ligand chains in dark blue as distinguished in **(C)** (top line).

Two proteins are presented as examples of disordered status recognition using the PONDR^®^ VL-XT program: hirudin and thrombin. The structure of these proteins is available in PDB 5HIR and 1NO9, respectively (Folkers et al., 1989; De Simone et al., 2003). The evaluation of these proteins from the point of view of the FOD model is given in Table 2; Figure 2.

**TABLE 2:** Characteristic of the complex (taken from Dyson and Wright (2002), being an example of using the PONDR analysis) according to the FOD model.

PDB	Complex		Individual chains				Chains as a part of the complex				Interface	Characteristics
	RD	K	RD	K	RD	K	RD	K	RD	K	RD	
PONDR analysis												
1NO9-H/L	0.313	0.0	0.326	0.0	0.311	0.0	0.321	0.1	0.428	0.0	0.316	SS: 0.227 (0.0), 0.303 (0.0), and 0.324 (0.1)
Chain N/I					0.265	0.0			0.240	0.0	0.339	Cat: 0.266 (0.1) and Cat + 5 0.231 (0.0)
5HIR			0.302	0.0								SS: 0.324 (0.0), 0.236 (0.0), and 0.267 (0.0)

In addition to the values of the RD and K parameters, a short characteristic is also given. SS—fragment of chain limited by Cys positions constructing SS bonds.

According to an analysis based on the identification of MoRFs, the complexation observed in the hirudin to prothrombin relationship represents an example of the involvement of IDR (hirudin C-terminal fragment) in an interaction that shows high ordering in target molecule alignment according to the PONDR analysis. Confronting these conclusions with the results based on the analysis of the structuring of the hydrophobic core, hirudin shows a very high adaptation of the hydrophobic distribution to the idealized distribution. Likewise, the entire complex (along with the interface fragments) exhibits a 3D Gaussian distribution of hydrophobicity. The C-terminal multi-amino acid fragment of hirudin (not present in the accessible structure) perhaps introduces local disorder and high flexibility. The values of K = 0.0 for both the complex and its components and low values of RD for the sections defined by appropriate disulfide bonds indicate high stabilization in the form of a highly ordered hydrophobic core supported by an appropriate arrangement of disulfide bonds.

This conclusion comes from the interpretation of RD and K values calculated for chain fragments determined by positions of Cys creating the SS bonds. The presence of a hydrophobic core and a system of disulfide bonds are generally treated as important factors for tertiary structure stabilization. The status of the hydrophobic core (accordant with the micelle-like organization) appears to be supported by the SS bond system since the fragments of chain defined by SS bond positions represent the status supporting the hydrophobic core construction (the low RD values calculated by fragments limited by Cys positions). The status of catalytic residues determined only for the residues involved in the catalytic activity of prothrombin and the immediate surroundings of these residues also shows a consistent hydrophobicity pattern with the idealized one. Summing up, from the point of view of FOD, the structure of hirudin and the complex in question is an example of a system highly representing the idealized system referred to as the hydrophobic core. This ordering applies to the centric concentration of hydrophobicity as well as the coating, with a decreasing level to the surface showing hydrophobicity close to zero. This is how the hydrophobic core is understood in terms of the FOD model (Figure 2).

### 2.3 Drug targets

**FIGURE 3:**
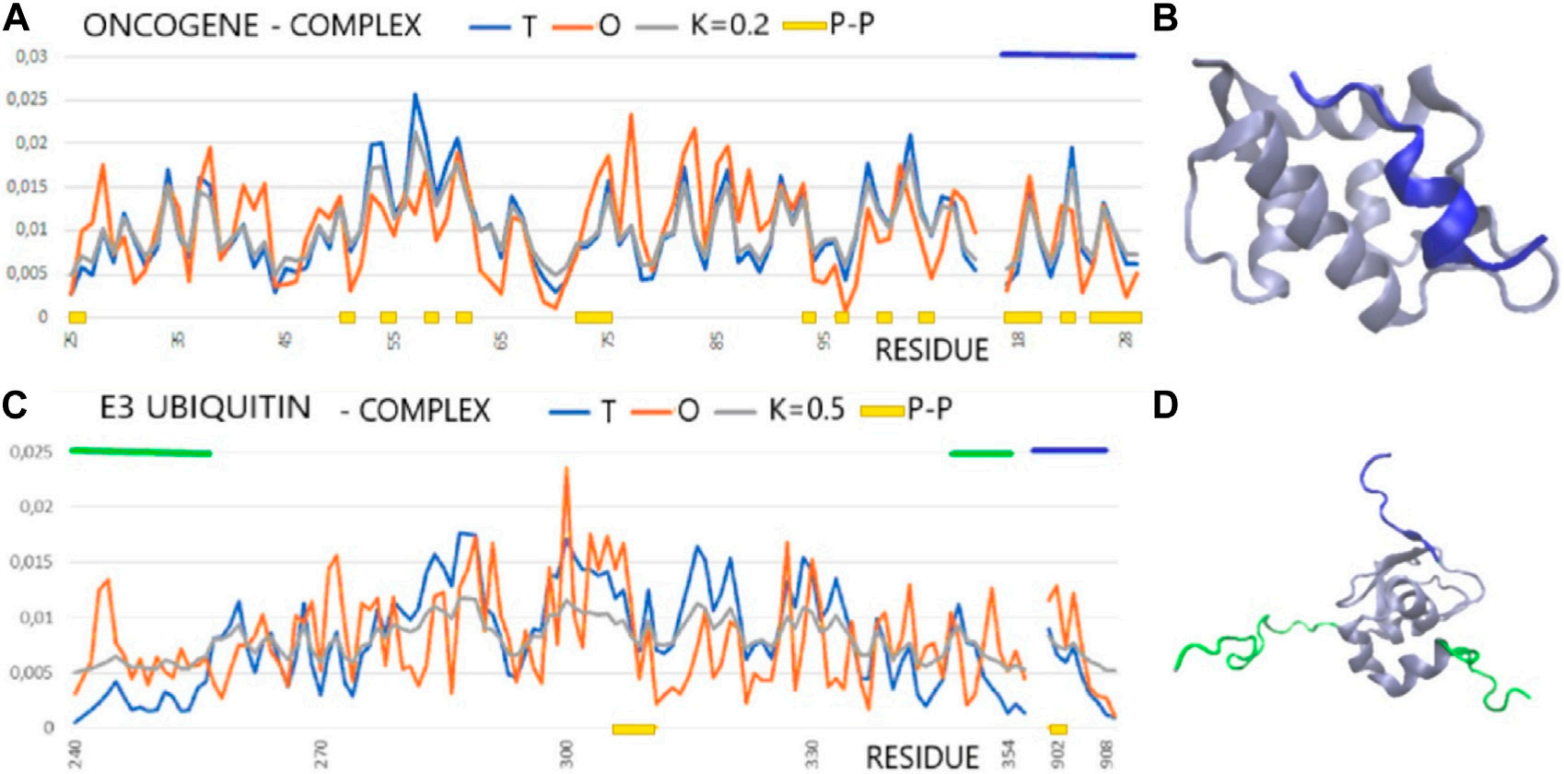
T, O, and M profiles for complexes (x-axis—positions of residues in the aa sequence in the chain, y-axis—hydrophobicity: T—blue, O—red): **(A)** The dark blue horizontal line on the top—ligand (PDB ID—1YCR). **(B)** 3D presentation of the 1YCR with ligand distinguished in dark blue [as in **(A)**]. **(C)** E3 ubiquitin (PDB ID—1G3F) dark blue horizontal line on top—ligand, green horizontal lines on top—N- and C-terminal fragments of loose structure eliminated in calculations discussed in the text. **(D)** 3D presentation of the 1G3F. The sections eliminated in the calculations as shown in Figure **(C)** are marked in green, red, and dark blue.

IDPs and IDRs also have a special place in the discussion on the search for new drugs, although the number of examples of their practical application is limited. Following the analysis given in Johannes et al. (2020), the status of both the target molecule and the form of the complex was determined for the 1YCR—complex E3 ubiquitin–protein ligase Mdm2 with fragment of p53 protein (Kussie et al., 1996). The 1BXL—Bcl-2-like protein 1 in complex with Bcl-2 homologous antagonist/killer (Sattler et al., 1997) shows, according to the FOD-based model analysis, a system with a high matching of the hydrophobicity distribution to the idealized hydrophobic core (Table 3; Figure 3).

**TABLE 3:** Summary of RD and K values describing the status of target proteins for new drugs.

PDB	Complex		Individual chains				Chains as a part of the complex				Interface	Characteristics
	RD	K	RD	K	RD	K	RD	K	RD	K	RD	
Drug targets												
1YCR	0.399	0.2	0.381	0.1	0.375	0.0	0.413	0.2	0.328	0.1	0.278	Inhibitor P53 helix
1BXL	0.406	0.2	0.415	0.2	0.247	0.0	0.406	0.2	0.404	0.0	0.406	20-residue helix of BAK HHH
1G3F	**0.578**	**0.5**	**0.616**	**0.6**	0.439	0.1	**0.595**	**0.5**	0.086	0.0	0.466	β-Strand fragment (AVPIAQKSE) of Smac BBBBBB
	0.467	0.3	0.497	0.4	0.439	0.1	0.493	0.3	0.096	0.0	0.403	Fragments 259–345

In the FOD-based assessment, it appears that the alignment of cellular tumor antigen p53 to n Mdm2 ligase (*Homo sapiens*) (1YCR) is based on the hydrophobicity ordering. The same occurs for Bcl-2-like protein 1 in complex with Bcl-2 homologous antagonist/killer (*Homo sapiens*) (1BXL). The situation is different in the case of E3 ubiquitin–protein ligase XIAP (inhibitor of apoptosis protein 3—*Homo sapiens*) in a complex with nine residue peptides from Smac/DIABLO. The target protein shows no structuralization based on a stable hydrophobic core, although the status of the complex is described by a RD value lower than that for the individual chain. The ligand match to the local order of the micelle-like kind seems to be perfect, which also expresses the low value of RD expressing the interface status. An exception to the abovementioned list is the complex 1G3F—E3 ubiquitin–protein ligase XIAP in the complex with Smac-promoting caspase activation (Liu et al., 2000). The structure of the target molecule chain is characterized by the presence of the N- and C-terminal fragments of a very loose structure with a clear disorder. These segments are not components of the globule, which is a large part of the chain. The elimination of these fragments from the analysis reveals the presence of a highly ordered hydrophobicity distribution in the complex and within its individual components, with the interface status highly suited to the idealized distribution (Figures 3B, C).

The factor of structural adjustment of the designed drug in the form of participation in the jointly constructed ordered distribution of hydrophobicity should be taken into account, thus assessing the obtained stabilization of the complex. Ensuring stabilization of the drug in the target protein structure may be a significant factor in obtaining a beneficial therapeutic effect. It was shown in the example of the proposed design of drugs to stop the propagation of amyloid fibrils (Roterman et al., 2018; Banach et al., 2020a).

### 2.4 Function-related unfolding

The examples discussed in this section have been taken from the work of Uversky (2013). Among the three structural forms of this protein: pG (ground state), pR (early intermediate state), and pB (transient signaling state), two structures are available: pG and pB (PDB ID 3PHY and 2KX6, respectively) (Düx et al., 1998; Ramachandran et al., 2011). Knowing them makes it possible to identify structural changes related to the function—in this case, structural changes resulting from activation due to exposure to blue light. Protein acts as a photo-sensor. The effect of photon absorption is a significant unfolding present in the pB state (Antes et al., 2002). Due to the significant reduction in the presence of secondary structure [β-structure—reduced to 18%, helical to 36% of the pG state—after PDBSUM (Laskowski et al., 2018)], this protein is discussed in the context of the DisProt issue (Chattopadhyaya et al., 1992; Lee et al., 2001; Liu et al., 2003; Yap et al., 2003; Dyer et al., 2004).

The analysis of the structuring of this protein from the point of view of the presence of a hydrophobic core, which is essential for the stabilization of all proteins, shows, in both forms, a significant degree of ordering of the hydrophobic distribution corresponding to the structure of the centric hydrophobic core. Low values of the RD (a RD significantly below the threshold value = 0.5), and especially a very low value of the parameter K = 0.2, indicate that the micelle-like structuralization is retained in both forms despite the significant degree of unfolding. This condition is important for the interpretation of protein characteristics (Table 4). On the one hand, the preservation of structuralization in the form of a centric concentration of hydrophobicity suggests that restoring the pG state is simple as a return to the structuring of the hydrophobic core, which, in fact, has not been eliminated. On the other hand, the low value of the K parameter indicates that the structure is dependent on the environment—especially in the absence of disulfide bonds in this protein. Changing the characteristics of the water environment to less polar or introducing a hydrophobic element in the environment of the discussed protein may significantly affect its structuring.

**TABLE 4:** Values of RD and K parameters describing the protein status in its two forms: ground state and function-related unfolding.

PDB	Complex		Individual chains				Chains as a part of the complex				Interface	Characteristics
	RD	K	RD	K	RD	K	RD	K	RD	K	RD	
Function												
3PHY			0.406	0.2								Basic structural form
2KX6			0.434	0.2								Function-related unfolding

**FIGURE 4:**
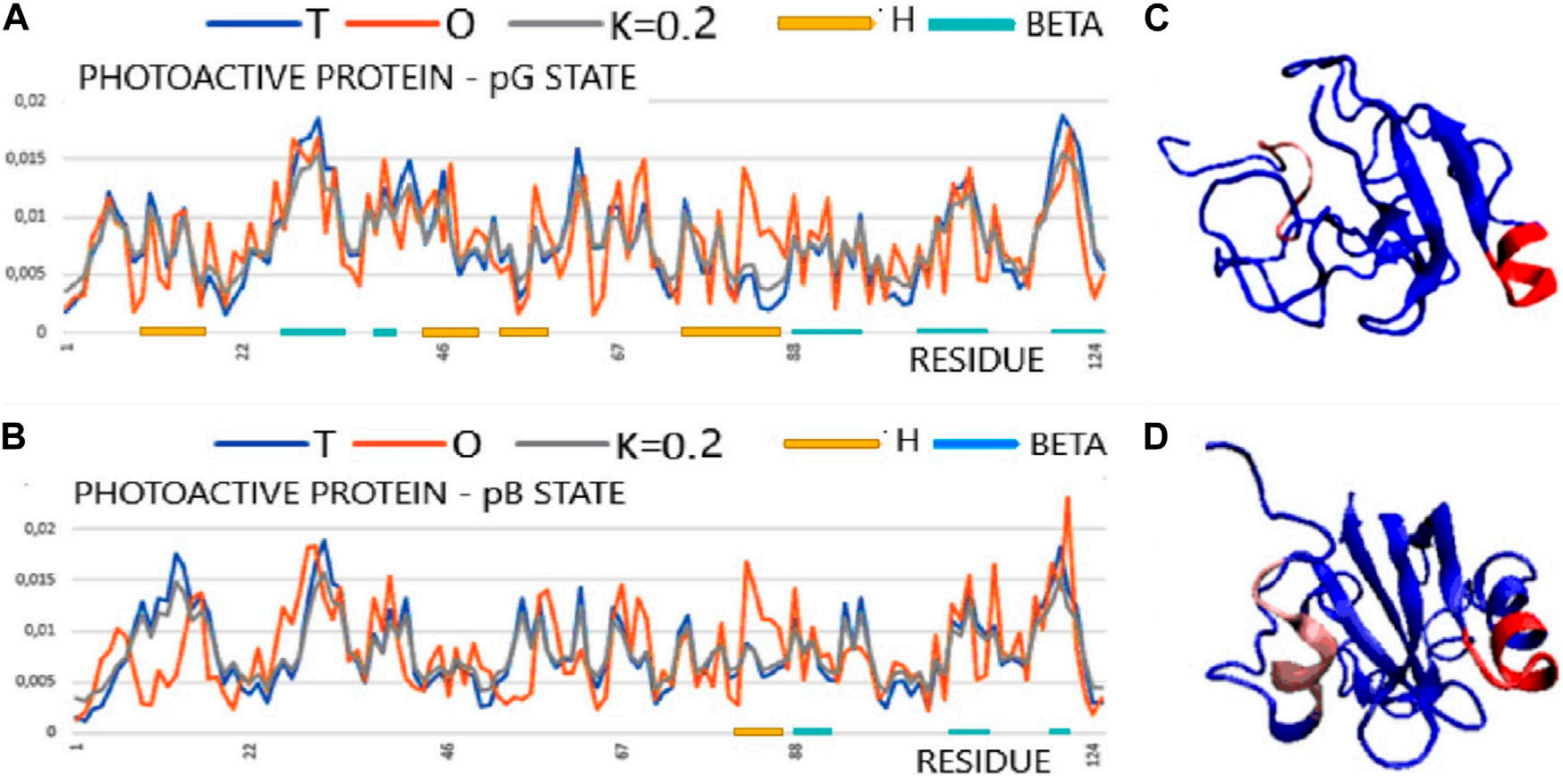
Structural characteristics of the PYP protein in the form of T, O, and M profiles. **(A)** Photoactive protein pG form (PDB ID 3PHY). **(B)** 3D presentation of photoactive protein pG form. **(C)** Photoactive protein pB form (PDB ID 2KX6). **(D)** 3D presentation of photoactive protein pB form. In graphs **(A)** and **(C)**, horizontal lines identify segments with a secondary structure: H—helix; beta—beta-structure. Sections 81–87 are highlighted in the 3D structure in both structural forms to show a local excess of hydrophobicity. The highlighted sections 8–15 (red) changed their status most to the structure of the hydrophobic core (pink) [according to profiles **(A)** and **(C)**].

The T, O, and M profiles (Figure 4) reveal a high degree of hydrophobicity ordering in relation to the idealized distribution with a negligible degree of modification of the target distribution for this protein in both structural forms. In Figure 4, the set of profiles is compared with the presentation of the 3D structure, indicating a significant degree of unfolding while maintaining the micelle-like pattern.

### 2.5 Unbound/bound

In analyzing protein complexation, it is important to compare the unbound structure with the bound form. The proteins described and classified with regard to the registered structural change (Table 5, right column) present here show various forms in the analysis presented here, using the criterion of participation in the structure of the hydrophobic core (Table 5).

**TABLE 5:** RD and K parameter values for protein pairs to identify bound and unbound form differences.

PDB	Complex		Individual chain				Chains as a part of the complex				Interface	Characteristics
	RD	K	RD	K	RD	K	RD	K	RD	K	RD	
Unbound–bound												
1U8T (Dyer et al., 2004)	0.420	0.3	0.378	0.2	0.326	0.1	0.372	0.2	**0.556**	0.4		D1: 0.448 (0.3) and D2: 0.425 (0.2)
												Unbound
												Small-scale
1F4V (Lee et al., 2001)	0.449	0.3	0.428	0.3	0.528	0.3	0.437	0.2	0.402	0.4	0.368	D1: 0.450 (0.3) and D2: 0.384 (0.2)
												Bound
												Small-scale
1CLL (Chattopadhyaya et al., 1992)			**0.718**	**2.0**								Unbound
			D1 0.450	**0.3**								Large-scale
			D2 0.384	**0.2**								
1NWD (Yap et al., 2003)	0.505	0.4	**0.544**	0.6	0.407	0.1	**0.533**	0.6	0.329	0.1	0.455	Bound BiC jako wspólny ligand
			D1 0.448	0.3								Large-scale tutaj calosc
			D2 0.426	0.2								
1PQ0 (Liu et al., 2003)			0.472	0.4							0.311	Unbound
												Partial order-to-disorder
1PQ1 (Liu et al., 2003)	0.369	0.2	0.407	0.3	0.711	0.9	0.357	0.2	0.474	0.3	0.340	Bound
												Partial order-to-disorder
1RWZ (Chapados et al., 2004)			**0.625**	**0.8**								D1: 0.458 (0.3) and D2: 0.478 (0.3)
												Unbound
												Partial disorder-to-order
1RXZ (Chapados et al., 2004)	**0.609**	**0.7**	**0.609**	**0.7**	0.187	0.0	**0.619**	**0.7**	0.382	0.0	0.434	D1: 0.436 (0.3) and D2: 0.477 (0.3)
												Bound
												Partial disorder-to-order

In proteins where domains are present, a set of domain parameter values is also given (denoted as D). Bold values—RD > 0.5—mean micelle-like disordering. Value in quotation marks—a value that expresses the status of the residues in the unbound protein that are part of the interface in the protein in the bound form. The “characteristics” column gives the classification of structural changes identified in Johannes et al. (2020).

**FIGURE 5:**
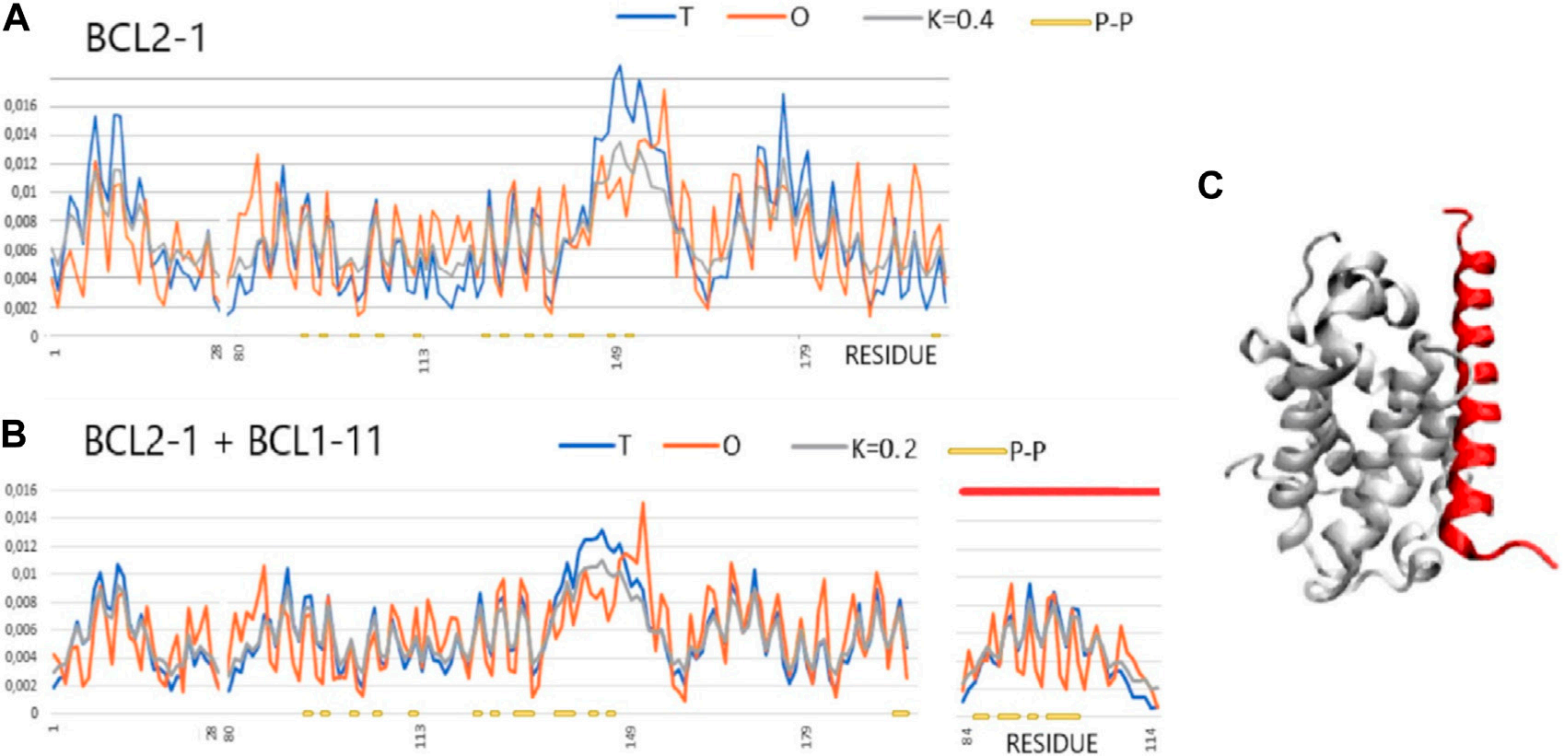
T, O, and M profiles for bound and unbound analyses (*Mus musculus*) (x-axis—positions of residues in the aa sequence in the chain, y-axis—hydrophobicity: T—blue, O—red). **(A)** Unbound Bcl-2-like protein 1 (1PQ0). **(B)** Bound to Bcl-2-like protein 11 (1PQ1). Lines (orange) on the horizontal axes indicate the residues involved in the P–P interaction. Ligand—red line on the top axis. **(C)** 3D presentation of the complex; red—complexed chain (Bcl-2-11) as shown in **(B)**.

The set of representatives of different states in the comparative analysis of the monomer and the complex form selected in the work of Dygut et al. (2016) shows the compliance of the interface status with the FOD model in all cases. This means that the very interaction of the target with the complexed molecule locally fits into the structure of the order consistent with the micelle-like form. The distinguished small-scale form is represented in light of the criteria related to fuzzy oil drop; the model shows the presence of a hydrophobic core in complexes as well as in an unbound form (1U8T/1F4V)—exodeoxyribonuclease/chemotaxis CheY protein (*Phage* t5i *E.coli*, respectively). This pair of proteins represents the one distinguished in Johannes et al. (2020), a group defined as “small-scale” as showing ordering according to the FOD model except for the status of complex units treated as components of the complex. The interface status in both cases shows local ordering according to an idealized micelle-like distribution. An example illustrating the “large-scale” structural alteration is calmodulin. It is an interesting subject for FOD-based analysis. A protein composed of two domains connected by a loose helical fragment radically changes its structure, turning into a complex. It is obvious that the hydrophobicity distribution is not ordered according to the 3D Gauss function in the situation of two domains that are distant from each other, which, however, as individual structural units, represent the hydrophobicity ordering according to the FOD model (low K values for both domains). After complex formation, its status is still expressed as a RD value greater than 0.5, although significantly lower than the free chain status. The domains in the complex also retain their ordering status according to the 3D Gauss function (Figure 5).

A representative group characterized as “partial order-to-disorder” is a set of two forms: 1PG0 and 1PQ1—apoptosis regulator bcl-x interacting with bcl2 interacting mediator of cell death (*Halorhodospira halophila*). This example shows an ordering consistent with the 3D Gauss distribution both in the complex form (including the components of this complex) and in the unbound form. Only the short-chain bcl2 interacting mediator of cell death treated as an individual structural unit shows a significant deviation from the order predicted by the FOD model. This means a significant degree of adaptation of this molecule to the target because, as a component of the complex, this molecule shows adaptation to the structure of a common hydrophobic core.

The pair of structures represented by the DNA polymerase sliding clamp (*Archaeoglobus fulgidus*) and its complex with flap endonuclease 1 qualified (PDB IDs 1RWZ and 1RXZ) (Chapados et al., 2004) as representing “partial disorder-to-order” shows the absence of hydrophobic core construction both in the free chain form and the complex. However, the interface status shows local hydrophobicity ordering consistent with the hydrophobic core design as predicted by the FOD model.

The comparison of 1PQ0 and 1PQ1 from the point of view of the FOD model may be an example of an interaction formed by two proteins (more precisely, a protein and a peptide) composed of two systems, both of which, in the unbound form, show the hydrophobic order in accordance with the 3D Gauss distribution.

**FIGURE 6:**
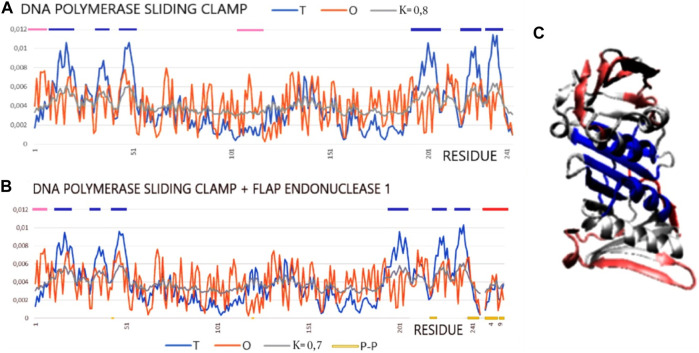
Profiles T, O, and M for DNA polymerase sliding clamp (*Archaeoglobus fulgidus*) in forms: **(A)** unbound (PDB ID—1RWZ); **(B)** bound to flap endonuclease 1 (red line on top) (*Archaeoglobus fulgidus*) (PDB ID—1RXZ). **(C)** 3D presentation with residues distinguished: excess of hydrophobicity—pink; hydrophobicity deficiency—dark blue, red chain—flap endonuclease 1. Residues representing hydrophobicity excess distinguished as dark blue lines on the top axis and residues representing hydrophobicity deficiency as pink lines on the top axis.

The examples of 1RXZ and 1RWZ proteins show the mismatch between the structure of the hydrophobic core and the expected 3D Gauss function distribution (Table 5; Figure 6). The reasons for the mismatch between the O distribution and the T distribution result from a significant deficit of hydrophobicity in the central part of the molecule. The local maxima in the T distribution are not duplicated in the O distribution. Moreover, an increased level of hydrophobicity on the surface is also visible. As a result, the distribution that most closely reproduces the O distribution is modified as far as K = 0.7 and K = 0.8. This means that both the unbound and bound structures of this protein were formed with a significantly reduced proportion of the polar aquatic environment, which neither led to the concentration of hydrophobic residues in the central part nor induced the exposure of polar residues on the surface. It seems to be an image of a molecule prepared to interact with DNA that requires adaptation to the structure of the nucleic acid and that requires the polymerase molecule to adapt to the sequence of nucleotides. Flap endonuclease 1 status, despite the short length of this peptide, shows the central location of the hydrophobic residues which, as shown by the value of RD for the interface status, adjust locally to the hydrophobicity distribution corresponding to the idealized distribution. A review of the numerous complexes of varying status, both monomers and dimers (and higher complexes), has been discussed. The differentiation observed also in the examples discussed in the works of Banach et al. (2020b) and Banach et al. (2020c) was demonstrated. The results described here confirm the template-dependent folding rather than the preformed elements model (Oldfield et al., 2007; Miskei et al., 2017). This is evidenced by the status of all discussed interfaces as locally adapted to the micelle-like distribution, which expresses the local adaptation of a given structure to the target molecule.

### 2.6 Disorder profile and functionality of p53

The p53 protein is critical in cancer research. It is a frequent subject of analysis due to its high medical importance. Point mutations present in its sequence are the cause of numerous cancers (Duffy et al., 2017). This protein is identified as an IDP (Signorelli et al., 2017). The results of the PONDR analysis (Dyson and Wright, 2002) show the different status of the sections of the protein chain in different complexes. Table 6 takes into account the division proposed in the work of Uversky (2011). Based on the results of the PONDR analysis, the terminal segment of the N-terminal fragment and the terminal fragment of the C-terminal fragment in the PONDR evaluation are indicated as IDR. Based on this analysis, the central fragments 95–290 were identified only locally over a relatively short distance as IDR.

**TABLE 6:** List of RD and K parameters characterizing the status of target proteins and fragments of the p53 chain complexed with them.

Disorder profile and functionality of p53												
p53 fragments 94–289												
	Complex		p53-individual		Target-individual		p53 in complex		Target in complex		Interface	Characteristics
1TSR (Cho et al., 1994)			**0.564**	**0.5**							**“0.689”**	K = 0.9—interaction with DNA
												Core domains 94–289
1GZH (Derbyshire et al., 2002)	**0.687**	**1.1**	0.499	0.4	0.409	0.2	**0.659**	**0.8**	**0.672**	**1.5**	0.457	Protection of telomeres protein 1 from *Schizosaccharomyces pombe* P53 + D1 chain B
1YCS (Gorina and Pavletich, 1996)	**0.719**	**1.3**	**0.582**	**0.6**	**0.554**	**0.5**	**0.731**	**1.0**	**0.703**	**1.5**	0.360	p53 residues 97–287
												(*Homo sapiens*) + 327–519 p53
2H1L (Lilyestrom et al., 2006)	**0.741**	**1.4**	**0.644**	**0.9**	**0.547**	**0.5**	**0.722**	**1.2**	**0.769**	**1.5**	**0.508**	Large T antigen
												(*Simian virus 40*)
												Chain C + chain O
Dom2	**0.715**	**1.1**	**0.520**	**0.5**	**0.547**	**0.5**	**0.740**	**1.8**	**0.654**	**0.7**	0.435	Dom2 chain A + chain C
p53C—terminal fragment												
3SAK (Kuszewski et al., 1999)			0.445	0.3								Monomers 1–42
	0.495	0.3	0.445	0.3	0.445	0.3	0.472	0.3	0.498	0.3	0.434	Cellular tumor antigen p53
												(*Homo sapiens*) 42 aa
												AB dimer
			0.445	0.3	0.445	0.3	0.493	0.3	**0.515**	0.4	**0.557**	AC dimer
1Q2D (Poux and Marmorstein, 2003)	0.383	0.2	0.373	0.2	0.359	0.0	0.369	0.2	**0.768**	**1.7**	0.414	19 aa p53 and 6 aa p53
1XQH (1XQHChuikov et al., 2004)	**0.619**	**0.7**	**0.620**	**0.7**	**0.821**	**0.4**	**0.635**	**0.7**	**0.336**	**0.1**	**0.682**	Histone–lysine N-methyltransferase SETD7 (*Homo sapiens*) + 6 aa p53
Dom	**0.524**	**0.4**	**0.519**	**0.4**	**0.821**	**0.4**	**0.527**	**0.4**	0.215	0.0	**0.645**	
1H26 (Lowe et al., 2002)	**0.534**	**0.5**	**0.531**	**0.5**	0.478	0.2	**0.542**	**0.2**	0.408	0.0	0.441	Cyclin-A2 (*Homo sapiens*) + **11 aa p53** chains B and E
Dome	0.301	0.1	0.307	0.1	0.478	0.2	0.306	0.1	0.342	0.0	0.371	Domain in chain B + **11 aa p53**
1MA3 (Avalos et al., 2002)	**0.527**	**0.5**	**0.543**	**0.5**	**0.671**	**0.8**	**0.535**	**0.5**	0.353	0.1	0.315	NAD-dependent protein deacylase 2 from (*Archaeoglobus fulgidus*) + 9 aa p53 **372–389**
Dom1	0.294	0.1	0.301	0.1	**0.671**	**0.8**	0.307	0.1	0.313	0.0	0.154	Domain in chain B
Dom2	0.400	0.1	0.433	0.1	**0.671**	**0.8**	0.419	0.1	**0.716**	**0.3**	0.254	Domain in chain B
1JSP (Mujtaba et al., 2004)	0.392	0.2	**0.503**	**0.4**	**0.731**	**0.8**	0.382	0.1	**0.749**	**1.0**	0.399	Bromodomain CREB-binding protein (*Homo sapiens*) + 20 aa p53
1DT7 (Rustandi et al., 2000)	0.426	0.2	0.419	0.2	**0.685**	**0.9**	0.396	0.2	**0.591**	**0.5**	0.421	22 aa
p53N—terminal fragment												
2GS0 (Di Lello et al., 2006a)	0.417	0.2	0.442	0.2	**0.527**	**0.3**	0.437	0.2	0.315	0.1	0.465	General transcription and DNA repair factor IIH subunit TFB1
												(*Saccharomyces cerevisiae*) + 14aap53
1YCR (Kussie et al., 1996)	0.400	0.2	0.381	0.1	0.375	0.0	0.413	0.2	0.328	0.1	0.278	E3 ubiquitin–protein ligase Mdm2 (*Homo sapiens*) + 13c aa p53
2B3G (Di Lello et al., 2006b)	0.393	0.2	0.315	0.1	**0.653**	**0.6**	0.398	0.2	**0.540**	**0.4**	**0.610**	Replication protein A 70 kDa DNA-binding subunit
												(*Homo sapiens*) + 24 aa p53

**FIGURE 7:**
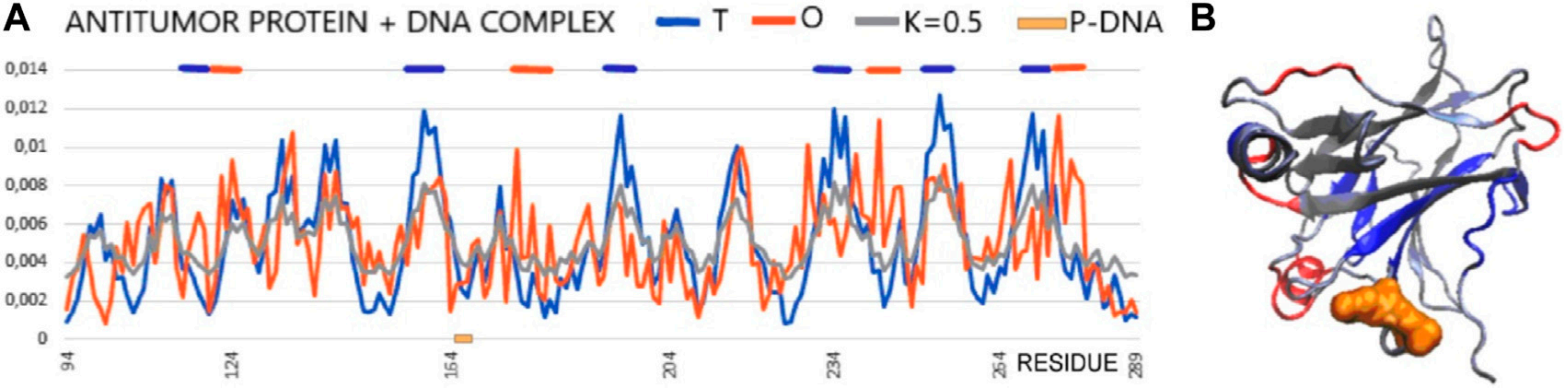
Core domain of p53 (PDB ID 1TSR). **(A)** T, O, and M profiles expressing the status of the p53 chain as identified in its complex with DNA. The helical section involved in interaction with DNA is distinguished on the horizontal axis (orange on the x-axis). **(B)** 3D presentation of the core domain of p53—DNA-binding protein. Red fragments identified as local deficiency according to profiles shown in **(A)**. Residues distinguished as shown in **(A)**. Residues representing local hydrophobicity deficiency—dark blue, local excess—red, interacting with DNA—orange space filling [as shown in **(A)**].

In the analysis presented here, the status of the chain of this protein available in PDB (1TSR 94–289), referred to as the core domain, shows the status expressed as RD > 0.5. hydrophobic core. This is illustrated by the set of profiles also revealing that this protein reproduces the structure of the hydrophobicity distribution modified with a value of K = 0.5. The main cause of non-compliance concerns the hydrophobicity deficit in the center (sections 155–160, 195–199, 230–238, 250–256, and 267–273) (Figure 7). It can be concluded that the core in this molecule does not meet the expectations as a structure stabilizer. There is also excess surface hydrophobicity in sections 104–107, 115–117, and 241–245, as well as in other short-surface sections (Figure 7). These differences, expressed by the value of RD, indicate a relatively loose structure with local redundant exposures of hydrophobicity. The position of the helix involved in the interaction with DNA, marked on the horizontal axis, obviously refers to the surface section, which also shows the level of hydrophobicity in this region, inconsistent with the idealized distribution.

Summarizing the assessment resulting from the FOD model-based analysis, this molecule does not have a hydrophobic core to ensure the stability of the molecule, which may prepare it to interact with a variety of targets to which it can adapt. This is visible in Figure 7, where the sections showing a local deficit are distinguished. This applies to centrally located chain fragments, which means rather poor stabilization resulting from the construction of the hydrophobic core. On the other hand, the short helical fragment exposed on the surface shows a locally higher polarity than it would appear from the 3D Gauss distribution.

The central section in the discussed examples of complexes shows a status with high values of RD and K. This means a structural deformation accompanied by a disruption of the structure of the hydrophobic core to a greater degree than in the isolated p53 molecule. This deformation mainly relates to the status of p53 as a component of the complex. High K values suggest a significant contribution of the target molecule, influencing the structuring of p53 to a great extent. However, the interface status (except for 2H1L) indicates that the ordering of the hydrophobicity level in this region from the point of view of the complex structure locally achieves the distribution expected in this region. The high values of RD and K for 1H1L may be due to the large size of the target molecule. Hence, the assessment of the status of the smaller molecule as a component of the complex (and the smaller molecule is p53) shows a mismatch with the idealized distribution (Miskei et al., 2017). The C-terminal segment shows the highest differentiation. This differentiation concerns both the status of the complexes and the status of individual structural units (treated as individual structural units and as components of complexes). High RD values appear with large-size target molecules. The different values of the K parameter reveal a different degree of structural adjustment of the p53 protein. Proteins in the complex with the N-terminal segment show the status relatively closest to the idealized distribution. Here, the values of RD < 0.5 prevail for both the status of the complexes and their components. The status of the interfaces except 2B3G shows RD < 0.5.

Summarizing the specificity of the p53 protein from the point of view of its systems in complexes with diverse target molecules, it can be stated that the protein represents adaptation possibilities in the form of adaptation to different targets (Cho et al., 1994; Derbyshire et al., 2002; Gorina and Pavletich, 1996; Lilyestrom et al., 2006; Kuszewski et al., 1999; Poux and Marmorstein, 2003; 1XQHChuikov et al., 2004; Lowe et al., 2002; Avalos et al., 2002; Mujtaba et al., 2004; Rustandi et al., 2000; Di Lello et al., 2006a; Di Lello et al., 2006b; Uversky, 2019). It can be speculated that it is a consequence of the relatively low share of the hydrophobic core in the stabilization of the structure of the discussed structural form of this protein, limited to the so-called core domain. Its characteristics are important because mutations in its area are most often observed in tumorigenesis processes (Duffy et al., 2017; Signorelli et al., 2017; Cho et al., 1994; Derbyshire et al., 2002; Gorina and Pavletich, 1996; Lilyestrom et al., 2006; Kuszewski et al., 1999; Poux and Marmorstein, 2003; 1XQHChuikov et al., 2004; Lowe et al., 2002; Avalos et al., 2002; Mujtaba et al., 2004; Rustandi et al., 2000; Di Lello et al., 2006a; Di Lello et al., 2006b; Uversky, 2019; Uversky, 2018; Bondos et al., 2021). The relation to the evaluation of this protein obtained in the PONDR analysis seems to be consistent with the evaluation presented here.

## 3 Discussion

Numerous studies on the role of IDPs and IDRs in biological activity indicate their significant importance in ensuring the functioning of this group of proteins. Taking into account the complexity of living organisms, and even single-cell organisms, it should be assumed that the activities of these organisms require the presence of various tools. Some of them are highly specific and determined, while others require flexibility to interact with diverse targets. For this purpose, tools with a flexible structure are needed and—as can be assumed—these are proteins with the status of IDPs or IDRs (Di Lello et al., 2006b).

The presence of a flexible structure is required primarily in tasks such as recognition, control, and regulation of their partners. Differentiation of signaling pathways requires the possible interaction with IDPs, allowing final fitting and stable interaction, making the expected biological function possible (Bondos et al., 2021; Fatafta et al., 2021). The presence of a flexible structure, however, creates conditions for incorrect inter-protein contacts, leading to pathological phenomena involving IDPs (ManiMishra et al., 2020; Bondos et al., 2021; Fatafta et al., 2021).

Proteins active in standard cellular conditions must adapt themselves to local environmental conditions, including solubility, which was shown by Sharma et al. (2019). The analysis proposed here supports this thesis by showing the presence of a significant portion of the IDP protein structure exhibiting a micelle-like character ensuring the solubility of the molecule despite the presence of a disordered region and even that of a 3D Gauss-type distribution concerning the whole protein if the IDP status is assigned to the whole chain.

The structures analyzed here are those stiffened by the presence of the target molecule. Not surprisingly, the status of the stretches involved in interactions in complexes—the interface component—of proteins with IDP status determined here shows it in some cases in accordance with other complexes of proteins not qualified as IDPs.

The presented model is focused on the hydrophobic interaction and hydrophobic-based distribution in complexes. The hydrophobicity is the object of other approaches as, for example, the prediction of trans-membrane proteins and the engagement of certain chain fragments in interaction with the hydrophobic environment present in the membrane (Kaburagi et al., 2007). The hydropathy index and formal charge of a test amino acid sequence using stochastic dynamical system models allow the identification of residues forming the trans-membrane part of proteins anchored in the cell membrane. The hydropathy index is successfully applied to identify the biological activity of proteins classified as unknown functions (Damodharan and Pattabhi, 2004). This issue is of special interest with respect to fast-growing databases collecting sequences of amino acids and 3D structures with missing information on biological activity. The hydropathy index is an important factor in large-scale analysis of proteins kingdom as it is used in MedProDB (Bhardwaj et al., 2021). The hydropathy index expresses the intrinsic specification of particular amino acid, while the Oi expresses the level of hydrophobicity as the effect of local influence of the surrounding, and thus it may be called the “effective” hydrophobicity level. Thus, it may be used to identify the local specificity in proteins as the synergy of hydropathy factors of close surroundings, in particular, of a 3D structure.

## 4 Materials and methods

### 4.1 Data

Table 7 summarizes the proteins analyzed in the present work. This summary was taken from the publications of van der Lee et al. (2014) and Sharma et al. (2019) after discussing the issues of IDPs in order to supplement the analysis with the assessment of the hydrophobic core status in this group of proteins.

**TABLE 7:** List of proteins present in the analysis together with their classification proposed in Li et al. (2000) and a short characteristic of biological activity.

PDB ID	Protein category	Ref
	MoRFs	
1X11	β-Amyloid precursor	Zhang et al. (1997)
1KY7	τ-MoRF	Brett et al. (2002)
1DP5	α-MoRF	Li et al. (2000)
1AVP	β-MoRF	Ding et al. (1996)
	PONDR	
1NO9	IDRs recognized	De Simone et al. (2003)
5HIR		Folkers et al. (1989)
	Drug targets	
1YCR	Inhibitor P53 helix	Kussie et al. (1996)
1BXL	20-residue helix of BAK HHH	Sattler et al. (1997)
1G3F	β-Strand fragment (AVPIAQKSE) of Smac	Liu et al. (2000)
	Function-related	
3PHY	Basic structural form	Düx et al. (1998)
2KX6	Function-related unfolding	Ramachandran et al. (2011)
	Bound–unbound	
1U8T	Unbound—small-scale	Dyer et al. (2004)
1F4V	Bound—small-scale	Lee et al. (2001)
1CLL	Unbound—large-scale	Chattopadhyaya et al. (1992)
1NWD	Bound—large scale	Yap et al. (2003)
1PQ0	Unbound—partial order-to-disorder	Liu et al. (2003)
1PQ1	Bound—partial order-to-disorder	Liu et al. (2003)
1RWZ	Unbound—partial disorder-to-order	Chapados et al. (2004)
1RXZ	Bound—partial disorder-to-order	Chapados et al. (2004)
	P53	
1TSR	Complex with DNA core domains 94–289	Cho et al. (1994)
1GZH	Protection of telomeres protein 1 from *Schizosaccharomyces pombe*	Derbyshire et al. (2002)
1YCS	p53 residues (*Homo sapiens*)	Gorina and Pavletich (1996)
2H1L		Lilyestrom et al. (2006)
3SAK	Monomers 1–42	Kuszewski et al. (1999)
1Q2D	19 aa p53 and 6 aa p53	Poux and Marmorstein (2003)
1XQH	Histone–lysine N-methyltransferase SETD7 (*Homo sapiens*) + 6 aa p53	1XQHChuikov et al. (2004)
1H26	Cyclin-A2 (*Homo sapiens*) + **11 aa p53** Chain B and E	Lowe et al. (2002)
1MA3	NAD-dependent protein deacylase 2 from (*Archaeoglobus fulgidus*) + 9 aa p53 **372–389**	Avalos et al. (2002)
1JSP	Bromodomain CREB-binding protein (*Homo sapiens*) + 20 aa p53	Mujtaba et al. (2004)
1DT7	22 aa	Rustandi et al. (2000)
2GS0	General transcription and DNA repair factor IIH subunit TFB1 (*Saccharomyces cerevisiae*)	Di Lello et al. (2006a)
1YCR	E3 ubiquitin–protein ligase Mdm2 (*Homo sapiens*) + 13c aa p53	Kussie et al. (1996)
2B3G	Replication protein A 70 kDa DNA-binding subunit (*Homo sapiens*) + 24 aa p53	Di Lello et al. (2006b)

### 4.2 Model description

The description of the model used for the analysis is included in Supplementary Material in order to avoid duplication of data contained in numerous works, including Roterman et al. (2022b).

The present work is aimed at determining the status of the sections identified as IDRs using a criterion related to the assessment of the participation of these sections in the structure of the hydrophobic core of proteins identified as IDPs.

### 4.3 Programs used

The potential used has two possible accesses to the program:

The program allowing calculation of the RD is accessible upon request on CodeOcean platform: https://codeocean.com/capsule/3084411/tree. Please contact the corresponding author to get access to your private program instance.

The application—implemented in collaboration with the Sano Center for Computational Medicine (https://sano.science) and running on resources contributed by ACC Cyfronet AGH (https://www.cyfronet.pl) in the framework of the PL-Grid Infrastructure (https://plgrid.pl)—provides a web wrapper for the abovementioned computational component and is freely available at https://hphob.sano.science.

The VMD program was used to present the 3D structures (Humphrey et al., 1996; Banach et al., 2021; VMD, 2021).

### 4.4 Calculation procedure

The applied calculation procedure is to determine the status of the chain, which includes the section identified as IDR. The characteristic is given by the RD parameter for the T–O–R relation and the value of the K parameter, which determines the degree of the proportion of a factor other than the aquatic environment. Additionally, the status of this segment IDR as a component of the structural unit is determined. If the chain has a domain structure, the structural unit against which the status of the IDR is determined is precisely the domain.

The assessment of a structural unit (chain/domain) from the point of view of the fuzzy oil drop model consists in generating a 3D Gaussian function encapsulating the entire unit. The value of the RD parameter determined with it defines the status as a whole. On the other hand, the status of a section classified as IDR consists in determining the contribution of a given section to the construction of the entire structural unit. In this situation, the selected fragment of T and O profiles, which was obtained for the entire unit, is subjected to normalization, and the RD value is determined. This value determines the share of a given segment in the structure of the centric hydrophobic core. For the normalized fragments of T and O profiles, the optimal K value is determined, which determines the degree of modification necessary to determine the status of a given fragment of the chain. The classification is common: RD < 0.5 both for the entire structural unit and for the selected section means participation in the structure of the hydrophobic core. Otherwise, the given unit does not show the presence of a hydrophobic core, and the section with such characteristics is treated as disturbing the system expected for the hydrophobic core.

## 5 Conclusion

The presented results of analyzes of proteins and their complexes classified as IDPs (or IDRs) using a non-geometric criterion for assessing their status reveal significant differences in terms of the participation of IDRs in the structure of the hydrophobic core. If we assume that the hydrophobic core is the stabilization factor of the tertiary structure, it turns out that the sections with intrinsically disordered forms in the complex arrangement reveal a fit to the hydrophobicity distribution in the micelle-like model. This means that their often geometrically disordered structure is subjected to adaptation to the expected idealized hydrophobicity distribution. This is evidenced by the low RD values for the interface zone. A protein representing function-related unfolding has a special place in the presented analysis. It has been shown that its significantly folded form (unfolding is accompanied by biological activity) retains the order with a centric hydrophobic core. It can be speculated that despite unfolding, the preserved presence of a centric concentration of hydrophobic residues allows this protein to return to its ground state structure. A similar situation, although concerning the structural changes of proteins undergoing amyloid transformation, was observed in the case of transthyretin [108]. Its form, aggressively undergoing this transformation after partial unfolding (the early intermediate model was used), acquires a structural form devoid of traces of a hydrophobic core. Another form of this protein—resistant to amyloid transformation—despite its partial unfolding (the same model was used), partially retains the concentration of hydrophobicity, which may allow it to return to its native form, thus preventing a permanent structural change [108].

Taking into account the frequently observed matching of IDPs and IDRs to the expected hydrophobicity distribution in the form of a centric hydrophobic core, it can be concluded that the proposed solution was confirmed in the results of this analysis (van der Lee et al., 2014).

## Data Availability

The datasets presented in this study can be found in online repositories. The names of the repository/repositories and accession number(s) can be found in the article/Supplementary Material.
