# Supplementary material for: Engagement of intrinsic disordered proteins in protein–protein interaction
Source: Front Mol Biosci. 2023 Jul 31;10:1230922. doi: 10.3389/fmolb.2023.1230922 (PMC10423874; doi:10.3389/fmolb.2023.1230922)
Supplement: Supplementary file 1 [file DataSheet1.pdf]

frontiers

*Supplementary Material*

# Engagement of intrinsic disordered proteins in protein-protein interaction

**Irena Roterman[1,]*, Katarzyna Stapor[2], Leszek Konieczny[3]**

*** Correspondence: Irena Roterman** : myroterm@cyf-kr.edu.pl

## Fuzzy oil drop model description

The short presentation of the model is given here to make easy interpretation of results presented in this paper [S1].

The majority of proteins are active in water environment. Treating the amino acids as bi-polar molecules makes possible to assume that they tend to construct the micelle-like structural form. The hydrophobic parts of side chains tend to concentrate in the central part with polar parts of amino acids direct toward surface. Thus the distribution of hydrophobicity may be expressed by 3D Gauss function spread all over the protein molecule.

$$T_i = \frac{1}{\sum_{i=1}^{N} T_i} \exp\left[\frac{-(x_i - \bar{x})^2}{2\sigma_x^2}\right] \exp\left[\frac{-(y_i - \bar{y})^2}{2\sigma_y^2}\right] \exp\left[\frac{-(z_i - \bar{z})^2}{2\sigma_z^2}\right] \qquad \text{eq.1.}$$

where $\sigma_x$, $\sigma_y$ and $\sigma_z$ are dependent on size and shape of the molecule.

The $T_i$ represents the idealized hydrophobicity level on the position of effective atom (averaged position of atoms belonging to particular amino acid).

The real observed hydrophobicity distribution however is the effect of inter-residual interaction expressed by Levitt's function [S2]

$$O_i = \frac{1}{\sum_{i=1}^{N} O_i} \sum_j \begin{cases} (H_i^r + H_j^r)(1 - \frac{1}{2}(1 - \frac{1}{2}(7(\frac{r_{ij}}{c})^2 - 9(\frac{r_{ij}}{c})^4 + 5(\frac{r_{ij}}{c})^6 - (\frac{r_{ij}}{c})^8 - (\frac{r_{ij}}{c})^8)), for r_{ij} \le c \\ 0, for r_{ij} > c \end{cases} \quad \text{eq.2.}$$

where $O_i$ represents the real level of hydrophobicity localized on the position of effective atom as the effect of hydrophobicity local collection. Any intrinsic hydrophobicity ($H^r$) scale of amino acids can be applied.

The two given distributions can be compared to measure the degree of similarity/differences between them using the convergence entropy introduced by Kullback-Leibler [S3]:

$$D_{KL}(O|T) = \sum_{i=1}^{N} O_i \log_2 \frac{O_i}{T_i} \quad \text{eq.3.}$$

The target - observed $O_i$ and reference distribution $T_i$ shall be normalized before the $D_{KL}$ can be calculated (the first component of eq. 1 and 2).

The value $D_{KL}$ as expressing entropy can not be interpreted. This is why the second reference distribution shall be introduced: $R_i = 1/N$ where N – number of residues in polypeptide chain. The R distribution is the opposite one to T distribution as representing the protein deprived of any form of hydrophobic core.

The T distribution in eq. 3 substituted by R distribution characterizes the differences between target distribution O and the reference distribution R.

The relation $D_{KL}(O|T) < D_{KL}(O|R)$ is interpreted as representing O distribution similar to T distribution thus the protein is classified as constructed according to micelle-like hydrophobicity distribution with hydrophobic core present.

To avoid operating with two parameters the Relative Distance (*RD*) is introduced as follows:

$$RD = \frac{D_{KL}(O|T)}{D_{KL}(O|T) + D_{KL}(O|R)} \quad \text{eq.4.}$$

The $RD < 0.5$ represents the protein with hydrophobic core.

*RD* values can be calculated for any structural unit: complex, single chain, domain. For each of these examples the 3D Gauss function is generated.

The status of selected fragments : chain in complex, domain in chain or even selected fragment of the chain in context of the distribution of selected unit can be assessed. In this case the sum of values of T*i*, O*i* and R*i* belonging to selected fragment shall be normalized before the *RD* can be calculated. In such case *RD* < 0.5 identifies the selected fragment as participating in the hydrophobic core construction or for *RD* > 0.5 introducing local disorder.

During folding process in water environment proteins accept the conformation directing the hydrophobic residues toward center of the molecule with exposition of hydrophilic residues on the surface. Sequence of amino acids in particular polypeptide chain allows the high-accordance micelle-like structuralization. Other sequence of amino acids in polypeptide chain allows the construction of micelle with local discordance in respect to micelle-like organization. These discordances appear to represent the localization of protein specificity. Local exposure of hydrophobicity on surface is very often used to construct the complex with other protein [S4]. The local hydrophobicity deficiency appears to represent the cavity – very often substrate binding cavity [S5]. The FOD model may be applied to proteins acting in other than water environment: in membrane for example [S6-S9]. The distribution of hydrophobicity in membrane proteins is opposite to that in water environment, what can be expressed by the following function:

$$M_i = (1 - T_i)_n \qquad \text{eq.5.}$$

where index „n” denotes normalization

In practice the following form is applied to calculate the opposite distribution:

$$M_i = (T_{MAX} - T_i)_n \qquad \text{eq.6.}$$

The analysis of numerous membrane proteins suggests the following function to be applied for description of hydrophobicity distribution in membrane proteins:

$$M_i = [T_i + K * (T_{MAX} - T_i)_n]_n \qquad \text{eq.7.}$$

where index „n” denotes normalization.

The interpretation of the eq. 7 is as follows:

The idealized distribution characteristic for water environment ($T_i$) gets modified by the opposite distribution ($T_{MAX}$-$T_i$) in the degree as measured by K parameter. This parameter expresses the degree to which the water directed force field gets modified by opposite force field (membrane based) to the extent measured by the K value.

The value of $D_{KL}(O|M)$ assess the proximity of O distribution in respect to M. The minimal value of $D_{KL}(O|M)$ for particular K value defines the best fit for O and M distributions. The K *($T_{MAX}$-$T_i$) expresses the degree in which the water derived force field gets modified by the external factor which is the membrane in particular.

The graphic presentation of the model shown in Fig. S1 is aimed to visualize the basic assumptions for FOD and FOD-M model.

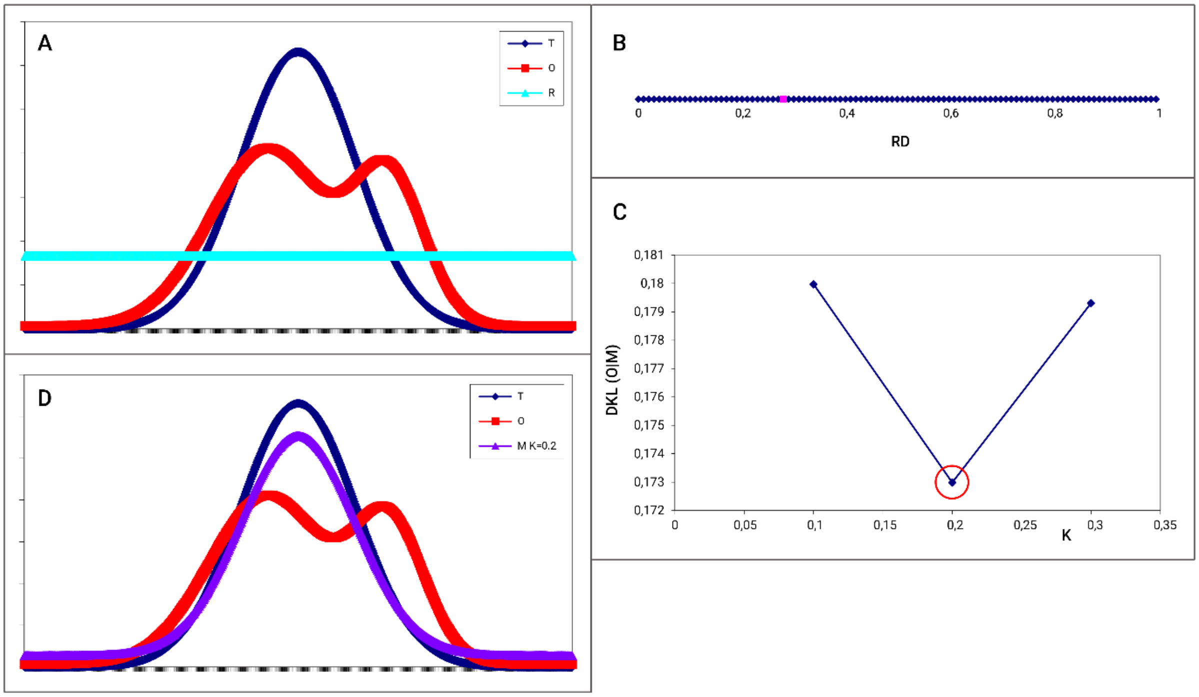


Fig. S1. Visualization of *K* value calculation.

A – *T*, *O* and *R* profiles (reduced to one dimension example)

B – *RD* value (0.27) for profiles as shown in A.

C – search for minimal $D_{KL}$ for *(O|M)* as dependent on *K*

D – *T*, *O* and *M* for *K*=0.2 as shown in C.

Status of individual unit assessment

The individual unit may be defined arbitrarily. The 3D Gauss function is constructed for one protein molecule, one chain isolated from complex, the complex as a whole. Parameters RD and K may be calculated for any structural unit.

Status of selected part of larger construction

The status of chain as the part of complex can be assessed in two ways: as integral part of complex – the 3D Gauss function constructed for all chains. This interpretation is focused on the analysis of the selected fragment of O profile in respect to T and R profile. The status of chain in complex can be estimated as integral part of T, O and R as well as M profiles.

However the status of chain can be treated as part of complex expressing its participation in hydrophobic core generation or introducing the local disorder (in the FOD interpretation as discordance of Oi status versus Ti status). To perform this analysis the selected fragments (for example chain in complex) of T, O and R profiles shell be normalized. The normalized Ti, Oi and Ri for selected fragment can be used for RD calculation expressing the status of selected fragment (chain in complex). This operation can be adopted for any fragment of chain chosen in particular construction. It reveals the participation of certain part of whole structure in core formation or introduction of local disorder. This procedure

The interpretation of *RD* and *K* parameter is as follows:

1. *RD* value characterize the order of hydrophobicity distribution in respect to idealized distribution. The higher *RD* the lower presence of hydrophobic core.
2. *K* value assess the degree to which the participation of other than water compounds directed the folding process. It is assumed that the environment directs this process toward organization accordant with external force field. The *K=0* characterizes the protein as representing micelle-like organization in water. Proteins as such have been recognized: down-hill proteins, fast-folding proteins, ultra-fast-folding and antifreeze proteins type II [S10]

**References:**

S1. Roterman I, Stapor K, Fabian P, Konieczny L, Banach M. Model of Environmental Membrane Field for Transmembrane Proteins. Int J Mol Sci. 2021; 22(7): 3619. doi: 10.3390/ijms22073619

S2. Levitt MA. A simplified representation of protein conformations for rapid simulation of protein folding. J. Mol. Biol. 1976; 104(1), 59-107. https://doi.org/10.1016/0022-2836(76)90004-8.

S3. Kullback S, Leibler RA. On information and sufficiency. Annals Mathemat Statistics 1951, 22(1), 79-86. https://doi.org/10.1214/aoms/1177729694.

S4. Dygut J, Kalinowska B, Banach M, Piwowar M, Konieczny L, Roterman I. Structural Interface Forms and Their Involvement in Stabilization of Multidomain Proteins or Protein Complexes *Int. J. Mol. Sci.* 2016, *17*(10), 1741; https://doi.org/10.3390/ijms17101741

S5. Banach M, Konieczny L, Roterman I. Ligand binding cavity encoded as a local hydrophobicity deficiency. In From globular proteins to amyloids. Ed. Irena Roterman-Konieczna, Elsevier 2020 Amsterdam Netherlands, Oxford UK, Cambridge MA USA, pp. 91-94.

S6. Roterman I, Stapor K, Gądek K, Gubała T, Nowakowski P, Fabian P, Konieczny L. Dependence of Protein Structure on Environment: FOD Model Applied to Membrane Proteins. Membranes (Basel). 2021; 12(1): 50. doi: 10.3390/membranes12010050

S7. Roterman I, Stapor K, Fabian P, Konieczny L. Connexins and Pannexins-Similarities and Differences According to the FOD-M Model. Biomedicines. 2022;10(7): 1504. doi: 10.3390/biomedicines10071504.

S8. Roterman I, Stapor K, Konieczny L. The Contribution of Hydrophobic Interactions to Conformational Changes of Inward/Outward Transmembrane Transport Proteins. Membranes (Basel). 2022; 12(12): 1212. doi: 10.3390/membranes12121212

S9. Roterman I, Stapor K, Fabian P, Konieczny L. The Functional Significance of Hydrophobic Residue Distribution in Bacterial Beta-Barrel Transmembrane Proteins. Membranes (Basel). 2021; 11(8): 580. doi: 10.3390/membranes11080580

S10. Banach M, Stapor K, Konieczny L, Fabian P, Roterman I. Downhill, Ultrafast and Fast Folding Proteins Revised. Int J Mol Sci **2020; 21(20): 7632.** doi: 10.3390/ijms21207632.
